# 1′-Methyl-4′-(4-methyl­phen­yl)dispiro­[1-benzopyran-3(4*H*),3′-pyrrolidine-2′,3′′-indoline]-2,2′′-dione

**DOI:** 10.1107/S1600536811051440

**Published:** 2011-12-03

**Authors:** D. Lakshmanan, S. Murugavel, D. Kannan, M. Bakthadoss

**Affiliations:** a Department of Physics, C. Abdul Hakeem College of Engineering & Technology, Melvisharam, Vellore 632 509, India; b Department of Physics, Thanthai Periyar Government Institute of Technology, Vellore 632 002, India; c Department of Organic Chemistry, University of Madras, Maraimalai Campus, Chennai 600 025, India

## Abstract

In the title compound, C_27_H_24_N_2_O_3_, the pyrroldine ring adopts a twist conformation, while the six-membered pyran­one ring of the coumarin ring system is in a sofa conformation. In the crystal, pairs of N—H⋯O hydrogen bonds link the mol­ecules into inversion *R*_2_^2^(8) dimers. These dimers are further connected *via* C—H⋯O hydrogen bonds.

## Related literature

For applications of pyrrolidine derivatives, see: Huryn *et al.* (1991 ▶); Suzuki *et al.* (1994 ▶); Waldmann (1995 ▶). For ring puckering parameters, see: Cremer & Pople (1975 ▶) and for asymmetry parameters, see: Duax *et al.* (1976 ▶). For closely related pyrrolidine structures, see: Selvanayagam *et al.* (2011 ▶); Ali *et al.* (2010 ▶). For hydrogen-bond motifs, see: Bernstein *et al.* (1995 ▶).
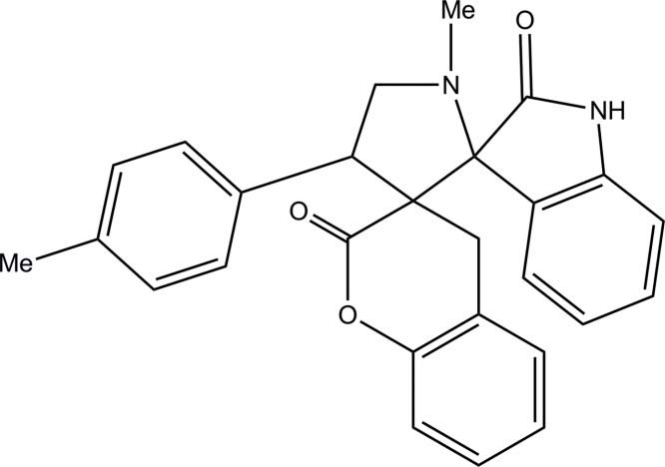


## Experimental

### 

#### Crystal data

C_27_H_24_N_2_O_3_
*M* *_r_* = 424.48
Monoclinic, 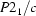

*a* = 10.4543 (3) Å
*b* = 14.6018 (4) Å
*c* = 14.7266 (4) Å
β = 104.043 (2)°
*V* = 2180.85 (10) Å^3^
*Z* = 4
Mo *K*α radiation
μ = 0.09 mm^−1^
*T* = 293 K
0.26 × 0.23 × 0.18 mm

#### Data collection

Bruker APEXII CCD diffractometer
Absorption correction: multi-scan (*SADABS*; Sheldrick, 1996 ▶) *T*_min_ = 0.978, *T*_max_ = 0.985
30221 measured reflections
7055 independent reflections
4544 reflections with *I* > 2σ(*I*)
*R*_int_ = 0.031

#### Refinement

*R*[*F* ^2^ > 2σ(*F* ^2^)] = 0.050
*wR*(*F* ^2^) = 0.153
*S* = 1.02
7055 reflections
291 parameters
H-atom parameters constrained
Δρ_max_ = 0.31 e Å^−3^
Δρ_min_ = −0.23 e Å^−3^

### 

Data collection: *APEX2* (Bruker, 2004 ▶); cell refinement: *APEX2* and *SAINT* (Bruker, 2004 ▶); data reduction: *SAINT* and *XPREP* (Bruker, 2004 ▶); program(s) used to solve structure: *SHELXS97* (Sheldrick, 2008 ▶); program(s) used to refine structure: *SHELXL97* (Sheldrick, 2008 ▶); molecular graphics: *ORTEP-3* (Farrugia, 1997 ▶); software used to prepare material for publication: *SHELXL97* and *PLATON* (Spek, 2009 ▶).

## Supplementary Material

Crystal structure: contains datablock(s) global, I. DOI: 10.1107/S1600536811051440/bt5735sup1.cif

Structure factors: contains datablock(s) I. DOI: 10.1107/S1600536811051440/bt5735Isup2.hkl

Additional supplementary materials:  crystallographic information; 3D view; checkCIF report

## Figures and Tables

**Table 1:** Hydrogen-bond geometry (Å, °)

*D*—H⋯*A*	*D*—H	H⋯*A*	*D*⋯*A*	*D*—H⋯*A*
N2—H2⋯O1^i^	0.86	2.02	2.874 (1)	174
C5—H5*B*⋯O3^ii^	0.96	2.59	3.407 (2)	143

Symmetry codes: (i) 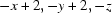; (ii) 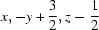.

**Fig. 1.**
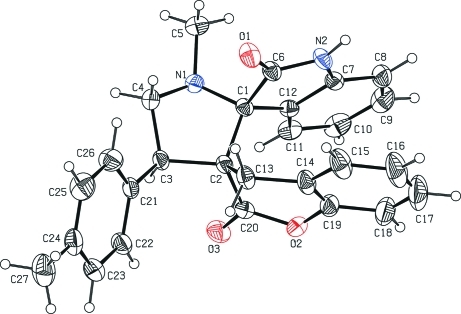
The molecular structure of the title compound with the atom numbering scheme. Displacement ellipsoids are drawn at the 30% probability level. H atoms are presented as a small cycles of arbitrary radius.

**Fig. 2.**
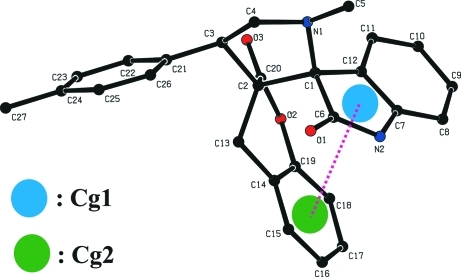
A view of the π···π interactions (dotted lines) in the molecular structure of the title compound. Cg1 and Cg2 are the centroids of the (N2/C1/C6/C7/C12) indole ring and (C14–C19) benzene ring, respectively.

**Fig. 3.**
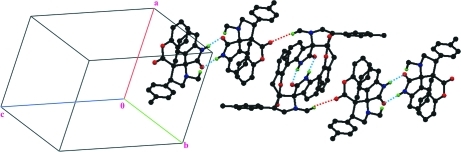
View of supramolecular zig zag chain in (I) with N—H···O (blue dashed lines) and C—H···O (red dashed lines) hydrogen bonds along the c axis.[ Colour code: O(red), N(blue), C(black) & H(green).

## supplementary crystallographic information

### Comment

Highly functionalized pyrrolidines have gained much interest in the past few years as they constitute the main structural element of many natural and synthetic pharmacologically active compounds (Waldmann, 1995). Optically active pyrrolidines have been used as intermediates, chiral ligands or auxiliaries in controlled asymmetric synthesis (Suzuki *et al.,* 1994; Huryn *et al.,* 1991). In view of this importance, the crystal structure of the title compound has been carried out and the results are presented here.

The title compound consists of a pyrrolidine ring connected to a oxindole ring system at C1, a coumarine moiety at C2 and a benzene ring at C3. The X-ray analysis confirms the molecular structure and atom connectivity as illustrated in Fig.1.

The pyrrolidine (N1/C1–C4) ring adopts a twist conformation , with twist about the C4—N1 bond; the puckering parameters (Cremer & Pople, 1975), q_2_ = 0.4216 (14) Å and φ_2_ = 156.9 (2)°, and asymmetry parameters (Duax *et al.,* 1976) ΔC_2_[C4—N1] = 5.0 Å. The six membered pyranone ring (O2/C2/C13/C14/C19/C20) of the coumarine moiety adopts screw-boat conformation as indicated from the puckering parameters: Q = 0.5296 (15) Å, θ = 65.9 (2)° and φ = 215.9 (2)°. The oxindole unit (N2/C1/C6–C12) is essentially planar [maximum deviation = 0.048 (1) Å for the C1 atom] and is oriented at a dihedral angles of 87.1 (1)° and 28.6 (1)°, respectively, with the pyrrolidine and coumarine rings. The sum of angles at N1 of the pyrrolidine ring (337°) is in accordance with *sp*^3^ hybridization, and the sum of angles at N2 of the indole moiety (360°) is in accordance with *sp*^2^ hybridization. The geometric parameters of the title molecule agrees well with those reported for similar structures (Selvanayagam *et al.*, 2011; Ali *et al.,* 2010).

Ihe molecular structure is stabilized by C3—H3···O3 and C13—H13A···O1 intramolecular hydrogen bonds, forming S(5) and S(6) ring motifs, respectively (Bernstein *et al.,* 1995) (Table 1). The molecular structure is further stabilized by an intramolecular π—π interactions with Cg1—Cg2 seperation of 3.539 (1) Å. (Fig. 2; Cg1 and Cg2 are the centroids of the (N2/C1/C6/C7/C12) indole ring, (C14–C19) benzene ring, respectively). The crystal packing is stabilized by intermolecular N—H···O and C—H···O hydrogen bonds. The molecules at *x, y, z* and *2-x, 2-y, -z* are linked by N2—H2···O1 hydrogen bonds into cyclic centrosymmetric *R_2_^2^*(8) dimers. This dimers are further connected by C5—H5B···O3 hydrogen bonds forming supramolecular zig zag chains along the *c* axis (Fig. 3).

### Experimental

A mixture of *E*-3-(4-methylbenzylidene)chroman-2-one (0.125 g, 0.5 mmol), isatin (0.08 g, 0.55 mmol) and *N*- methylglycine (0.025 g, 0.55 mmol) in toluene (5 ml) as solvent was allowed to reflux for 6 hours. After work up, the crude mass was purified by column chromatography to yield the pure product (0.195 g, 92% yield). The compound was recrystallized from ethyl acetate solvent. Single crystals suitable for X-ray diffraction were obtained by slow evaporation of a ethylacetate solution at room temperature.

### Refinement

H atoms were positioned geometrically, with N—H = 0.86 Å and and C—H = 0.93–0.98 Å and constrained to ride on their parent atom, with *U*_iso_(H)=1.5*U*_eq_ for methyl H atoms and 1.2*U*_eq_(C) for other H atoms.

### Crystal data

**Table:**

C_27_H_24_N_2_O_3_	*F*(000) = 896
*M**_r_* = 424.48	*D*_x_ = 1.293 Mg m^−^^3^
Monoclinic, *P*2_1_/*c*	Mo *K*α radiation, λ = 0.71073 Å
Hall symbol: -P 2ybc	Cell parameters from 7063 reflections
*a* = 10.4543 (3) Å	θ = 2.0–31.2°
*b* = 14.6018 (4) Å	µ = 0.09 mm^−^^1^
*c* = 14.7266 (4) Å	*T* = 293 K
β = 104.043 (2)°	Block, colourless
*V* = 2180.85 (10) Å^3^	0.26 × 0.23 × 0.18 mm
*Z* = 4	

### Data collection

**Table:**

Bruker APEXII CCD diffractometer	7055 independent reflections
Radiation source: fine-focus sealed tube	4544 reflections with *I* > 2σ(*I*)
graphite	*R*_int_ = 0.031
Detector resolution: 10.0 pixels mm^-1^	θ_max_ = 31.2°, θ_min_ = 2.0°
ω scans	*h* = −15→14
Absorption correction: multi-scan (*SADABS*; Sheldrick, 1996)	*k* = −21→21
*T*_min_ = 0.978, *T*_max_ = 0.985	*l* = −20→21
30221 measured reflections	

### Refinement

**Table:**

Refinement on *F*^2^	Primary atom site location: structure-invariant direct methods
Least-squares matrix: full	Secondary atom site location: difference Fourier map
*R*[*F*^2^ > 2σ(*F*^2^)] = 0.050	Hydrogen site location: inferred from neighbouring sites
*wR*(*F*^2^) = 0.153	H-atom parameters constrained
*S* = 1.02	*w* = 1/[σ^2^(*F*_o_^2^) + (0.0694*P*)^2^ + 0.398*P*] where *P* = (*F*_o_^2^ + 2*F*_c_^2^)/3
7055 reflections	(Δ/σ)_max_ < 0.001
291 parameters	Δρ_max_ = 0.31 e Å^−^^3^
0 restraints	Δρ_min_ = −0.23 e Å^−^^3^

### Special details

**Table:**

Geometry. All esds (except the esd in the dihedral angle between two l.s. planes) are estimated using the full covariance matrix. The cell esds are taken into account individually in the estimation of esds in distances, angles and torsion angles; correlations between esds in cell parameters are only used when they are defined by crystal symmetry. An approximate (isotropic) treatment of cell esds is used for estimating esds involving l.s. planes.
Refinement. Refinement of F^2^ against ALL reflections. The weighted R-factor wR and goodness of fit S are based on F^2^, conventional R-factors R are based on F, with F set to zero for negative F^2^. The threshold expression of F^2^ > 2sigma(F^2^) is used only for calculating R-factors(gt) etc. and is not relevant to the choice of reflections for refinement. R-factors based on F^2^ are statistically about twice as large as those based on F, and R- factors based on ALL data will be even larger.

### Fractional atomic coordinates and isotropic or equivalent isotropic displacement parameters (Å^2^)

**Table:**

	*x*	*y*	*z*	*U*_iso_*/*U*_eq_	
C1	0.80511 (12)	0.84780 (8)	0.09959 (8)	0.0326 (2)	
C2	0.80733 (12)	0.86879 (8)	0.20643 (8)	0.0335 (2)	
C3	0.65980 (12)	0.89026 (9)	0.20385 (9)	0.0367 (3)	
H3	0.6207	0.8333	0.2197	0.044*	
C4	0.59877 (13)	0.90885 (11)	0.10112 (10)	0.0449 (3)	
H4A	0.5042	0.8989	0.0858	0.054*	
H4B	0.6166	0.9709	0.0841	0.054*	
C5	0.63008 (17)	0.84632 (12)	−0.04637 (10)	0.0548 (4)	
H5A	0.5385	0.8313	−0.0695	0.082*	
H5B	0.6830	0.8033	−0.0705	0.082*	
H5C	0.6459	0.9070	−0.0662	0.082*	
C6	0.87724 (13)	0.92422 (9)	0.05686 (9)	0.0383 (3)	
C7	0.97873 (13)	0.79028 (9)	0.03856 (9)	0.0382 (3)	
C8	1.06331 (16)	0.72827 (11)	0.01407 (11)	0.0528 (4)	
H8	1.1311	0.7470	−0.0125	0.063*	
C9	1.04343 (19)	0.63686 (12)	0.03057 (13)	0.0626 (5)	
H9	1.1002	0.5932	0.0161	0.075*	
C10	0.94146 (19)	0.60908 (10)	0.06787 (12)	0.0586 (4)	
H10	0.9291	0.5469	0.0767	0.070*	
C11	0.85666 (16)	0.67219 (9)	0.09259 (10)	0.0455 (3)	
H11	0.7871	0.6532	0.1172	0.055*	
C12	0.87840 (13)	0.76409 (8)	0.07963 (8)	0.0348 (3)	
C13	0.90158 (13)	0.94596 (10)	0.25105 (10)	0.0450 (3)	
H13A	0.8788	1.0018	0.2152	0.054*	
H13B	0.8931	0.9573	0.3142	0.054*	
C14	1.04058 (14)	0.91967 (12)	0.25330 (11)	0.0520 (4)	
C15	1.13597 (18)	0.97997 (16)	0.23772 (14)	0.0717 (6)	
H15	1.1152	1.0414	0.2254	0.086*	
C16	1.2617 (2)	0.9486 (2)	0.24059 (18)	0.0957 (8)	
H16	1.3259	0.9893	0.2313	0.115*	
C17	1.2920 (2)	0.8584 (2)	0.2570 (2)	0.1000 (9)	
H17	1.3766	0.8380	0.2578	0.120*	
C18	1.19962 (18)	0.79673 (18)	0.27257 (14)	0.0791 (6)	
H18	1.2205	0.7352	0.2840	0.095*	
C19	1.07479 (14)	0.82966 (13)	0.27055 (10)	0.0544 (4)	
C20	0.85238 (14)	0.78283 (10)	0.26401 (9)	0.0418 (3)	
C21	0.63774 (12)	0.96203 (9)	0.27238 (10)	0.0395 (3)	
C22	0.64635 (16)	0.93919 (11)	0.36474 (11)	0.0509 (4)	
H22	0.6657	0.8791	0.3843	0.061*	
C23	0.62671 (17)	1.00382 (13)	0.42869 (12)	0.0584 (4)	
H23	0.6352	0.9864	0.4906	0.070*	
C24	0.59504 (15)	1.09288 (12)	0.40314 (13)	0.0560 (4)	
C25	0.5856 (2)	1.11562 (12)	0.31114 (15)	0.0677 (5)	
H25	0.5637	1.1754	0.2915	0.081*	
C26	0.60777 (18)	1.05209 (11)	0.24708 (12)	0.0576 (4)	
H26	0.6024	1.0703	0.1858	0.069*	
C27	0.5729 (2)	1.16348 (15)	0.47218 (17)	0.0806 (6)	
H27A	0.6150	1.2198	0.4624	0.121*	
H27B	0.6095	1.1420	0.5347	0.121*	
H27C	0.4800	1.1737	0.4635	0.121*	
N1	0.66462 (11)	0.84226 (8)	0.05524 (8)	0.0395 (2)	
N2	0.97478 (11)	0.88502 (8)	0.02528 (8)	0.0430 (3)	
H2	1.0282	0.9148	0.0000	0.052*	
O1	0.84748 (11)	1.00547 (6)	0.05046 (8)	0.0525 (3)	
O2	0.98473 (10)	0.76670 (8)	0.29036 (7)	0.0546 (3)	
O3	0.78215 (12)	0.72821 (8)	0.28723 (8)	0.0607 (3)	

### Atomic displacement parameters (Å^2^)

**Table:**

	*U*^11^	*U*^22^	*U*^33^	*U*^12^	*U*^13^	*U*^23^
C1	0.0345 (6)	0.0302 (6)	0.0358 (6)	−0.0025 (4)	0.0135 (5)	0.0040 (4)
C2	0.0307 (6)	0.0370 (6)	0.0351 (6)	−0.0028 (5)	0.0122 (5)	0.0013 (5)
C3	0.0304 (6)	0.0405 (6)	0.0426 (7)	−0.0013 (5)	0.0154 (5)	0.0039 (5)
C4	0.0343 (6)	0.0549 (8)	0.0459 (7)	0.0033 (6)	0.0108 (6)	0.0029 (6)
C5	0.0553 (9)	0.0659 (10)	0.0397 (7)	0.0022 (7)	0.0051 (6)	0.0038 (7)
C6	0.0412 (7)	0.0345 (6)	0.0434 (7)	−0.0018 (5)	0.0184 (5)	0.0055 (5)
C7	0.0409 (7)	0.0403 (7)	0.0343 (6)	0.0031 (5)	0.0110 (5)	−0.0006 (5)
C8	0.0489 (8)	0.0610 (10)	0.0504 (8)	0.0121 (7)	0.0157 (7)	−0.0087 (7)
C9	0.0666 (11)	0.0519 (9)	0.0654 (10)	0.0217 (8)	0.0080 (9)	−0.0158 (8)
C10	0.0752 (11)	0.0342 (7)	0.0583 (9)	0.0090 (7)	0.0007 (8)	−0.0044 (6)
C11	0.0564 (8)	0.0341 (6)	0.0432 (7)	−0.0043 (6)	0.0066 (6)	0.0020 (5)
C12	0.0406 (6)	0.0319 (6)	0.0315 (6)	0.0005 (5)	0.0079 (5)	0.0013 (4)
C13	0.0368 (7)	0.0510 (8)	0.0505 (8)	−0.0090 (6)	0.0172 (6)	−0.0151 (6)
C14	0.0345 (7)	0.0732 (10)	0.0499 (8)	−0.0107 (7)	0.0131 (6)	−0.0233 (7)
C15	0.0484 (9)	0.0934 (14)	0.0787 (12)	−0.0280 (9)	0.0260 (9)	−0.0347 (10)
C16	0.0436 (10)	0.144 (2)	0.1069 (18)	−0.0334 (13)	0.0318 (11)	−0.0466 (17)
C17	0.0351 (9)	0.155 (3)	0.1101 (19)	−0.0022 (13)	0.0174 (10)	−0.0376 (18)
C18	0.0427 (9)	0.1165 (17)	0.0735 (12)	0.0177 (10)	0.0055 (8)	−0.0151 (12)
C19	0.0348 (7)	0.0841 (12)	0.0422 (8)	0.0019 (7)	0.0050 (6)	−0.0116 (7)
C20	0.0441 (7)	0.0485 (8)	0.0344 (6)	0.0056 (6)	0.0126 (5)	0.0054 (5)
C21	0.0312 (6)	0.0437 (7)	0.0471 (7)	0.0024 (5)	0.0159 (5)	0.0019 (5)
C22	0.0566 (9)	0.0508 (8)	0.0516 (8)	0.0114 (7)	0.0253 (7)	0.0060 (6)
C23	0.0592 (10)	0.0708 (11)	0.0512 (9)	0.0109 (8)	0.0248 (8)	−0.0019 (8)
C24	0.0411 (8)	0.0614 (10)	0.0685 (10)	0.0054 (7)	0.0190 (7)	−0.0145 (8)
C25	0.0757 (12)	0.0464 (9)	0.0819 (13)	0.0145 (8)	0.0208 (10)	−0.0001 (8)
C26	0.0701 (11)	0.0485 (9)	0.0561 (9)	0.0107 (7)	0.0189 (8)	0.0069 (7)
C27	0.0697 (12)	0.0794 (14)	0.0981 (16)	0.0079 (10)	0.0307 (11)	−0.0328 (11)
N1	0.0348 (5)	0.0465 (6)	0.0364 (5)	−0.0024 (4)	0.0073 (4)	0.0027 (4)
N2	0.0459 (6)	0.0404 (6)	0.0509 (7)	−0.0014 (5)	0.0274 (5)	0.0064 (5)
O1	0.0583 (6)	0.0341 (5)	0.0744 (7)	0.0032 (4)	0.0344 (5)	0.0138 (5)
O2	0.0464 (6)	0.0712 (7)	0.0436 (5)	0.0159 (5)	0.0056 (4)	0.0086 (5)
O3	0.0682 (7)	0.0571 (7)	0.0645 (7)	0.0054 (5)	0.0314 (6)	0.0256 (5)

### Geometric parameters (Å, °)

**Table:**

C1—N1	1.4577 (16)	C13—H13A	0.9700
C1—C12	1.5089 (17)	C13—H13B	0.9700
C1—C6	1.5621 (16)	C14—C19	1.370 (3)
C1—C2	1.5975 (17)	C14—C15	1.391 (2)
C2—C20	1.5240 (18)	C15—C16	1.383 (3)
C2—C13	1.5356 (18)	C15—H15	0.9300
C2—C3	1.5652 (17)	C16—C17	1.363 (4)
C3—C21	1.5110 (18)	C16—H16	0.9300
C3—C4	1.5169 (19)	C17—C18	1.380 (4)
C3—H3	0.9800	C17—H17	0.9300
C4—N1	1.4497 (18)	C18—C19	1.384 (2)
C4—H4A	0.9700	C18—H18	0.9300
C4—H4B	0.9700	C19—O2	1.396 (2)
C5—N1	1.4529 (18)	C20—O3	1.1892 (17)
C5—H5A	0.9600	C20—O2	1.3637 (17)
C5—H5B	0.9600	C21—C26	1.382 (2)
C5—H5C	0.9600	C21—C22	1.382 (2)
C6—O1	1.2243 (16)	C22—C23	1.383 (2)
C6—N2	1.3467 (17)	C22—H22	0.9300
C7—C8	1.3736 (19)	C23—C24	1.372 (2)
C7—C12	1.3857 (18)	C23—H23	0.9300
C7—N2	1.3964 (18)	C24—C25	1.375 (3)
C8—C9	1.381 (2)	C24—C27	1.505 (2)
C8—H8	0.9300	C25—C26	1.382 (2)
C9—C10	1.374 (3)	C25—H25	0.9300
C9—H9	0.9300	C26—H26	0.9300
C10—C11	1.387 (2)	C27—H27A	0.9600
C10—H10	0.9300	C27—H27B	0.9600
C11—C12	1.3820 (18)	C27—H27C	0.9600
C11—H11	0.9300	N2—H2	0.8600
C13—C14	1.496 (2)		
			
N1—C1—C12	111.83 (10)	C2—C13—H13B	109.7
N1—C1—C6	113.05 (10)	H13A—C13—H13B	108.2
C12—C1—C6	100.52 (9)	C19—C14—C15	118.22 (16)
N1—C1—C2	102.96 (9)	C19—C14—C13	117.33 (14)
C12—C1—C2	117.43 (10)	C15—C14—C13	124.44 (17)
C6—C1—C2	111.50 (10)	C16—C15—C14	120.0 (2)
C20—C2—C13	106.66 (11)	C16—C15—H15	120.0
C20—C2—C3	110.37 (10)	C14—C15—H15	120.0
C13—C2—C3	112.93 (10)	C17—C16—C15	120.2 (2)
C20—C2—C1	108.58 (10)	C17—C16—H16	119.9
C13—C2—C1	114.63 (10)	C15—C16—H16	119.9
C3—C2—C1	103.64 (9)	C16—C17—C18	121.2 (2)
C21—C3—C4	116.52 (11)	C16—C17—H17	119.4
C21—C3—C2	115.59 (10)	C18—C17—H17	119.4
C4—C3—C2	103.51 (10)	C17—C18—C19	117.7 (2)
C21—C3—H3	106.9	C17—C18—H18	121.2
C4—C3—H3	106.9	C19—C18—H18	121.2
C2—C3—H3	106.9	C14—C19—C18	122.66 (18)
N1—C4—C3	102.26 (11)	C14—C19—O2	120.77 (13)
N1—C4—H4A	111.3	C18—C19—O2	116.53 (18)
C3—C4—H4A	111.3	O3—C20—O2	117.19 (13)
N1—C4—H4B	111.3	O3—C20—C2	125.69 (13)
C3—C4—H4B	111.3	O2—C20—C2	117.12 (12)
H4A—C4—H4B	109.2	C26—C21—C22	116.83 (14)
N1—C5—H5A	109.5	C26—C21—C3	122.78 (13)
N1—C5—H5B	109.5	C22—C21—C3	120.39 (12)
H5A—C5—H5B	109.5	C21—C22—C23	121.36 (15)
N1—C5—H5C	109.5	C21—C22—H22	119.3
H5A—C5—H5C	109.5	C23—C22—H22	119.3
H5B—C5—H5C	109.5	C24—C23—C22	121.72 (16)
O1—C6—N2	125.79 (12)	C24—C23—H23	119.1
O1—C6—C1	125.78 (11)	C22—C23—H23	119.1
N2—C6—C1	108.39 (11)	C23—C24—C25	117.01 (15)
C8—C7—C12	122.44 (13)	C23—C24—C27	122.04 (18)
C8—C7—N2	127.98 (13)	C25—C24—C27	120.95 (18)
C12—C7—N2	109.56 (11)	C24—C25—C26	121.77 (16)
C7—C8—C9	117.18 (16)	C24—C25—H25	119.1
C7—C8—H8	121.4	C26—C25—H25	119.1
C9—C8—H8	121.4	C21—C26—C25	121.29 (16)
C10—C9—C8	121.34 (15)	C21—C26—H26	119.4
C10—C9—H9	119.3	C25—C26—H26	119.4
C8—C9—H9	119.3	C24—C27—H27A	109.5
C9—C10—C11	121.08 (15)	C24—C27—H27B	109.5
C9—C10—H10	119.5	H27A—C27—H27B	109.5
C11—C10—H10	119.5	C24—C27—H27C	109.5
C12—C11—C10	118.15 (15)	H27A—C27—H27C	109.5
C12—C11—H11	120.9	H27B—C27—H27C	109.5
C10—C11—H11	120.9	C4—N1—C5	115.16 (11)
C11—C12—C7	119.70 (12)	C4—N1—C1	107.14 (10)
C11—C12—C1	130.64 (12)	C5—N1—C1	115.52 (11)
C7—C12—C1	109.60 (10)	C6—N2—C7	111.83 (11)
C14—C13—C2	109.89 (12)	C6—N2—H2	124.1
C14—C13—H13A	109.7	C7—N2—H2	124.1
C2—C13—H13A	109.7	C20—O2—C19	121.05 (12)
C14—C13—H13B	109.7		
			
N1—C1—C2—C20	107.25 (11)	C13—C14—C15—C16	−179.46 (17)
C12—C1—C2—C20	−16.07 (14)	C14—C15—C16—C17	1.2 (3)
C6—C1—C2—C20	−131.25 (11)	C15—C16—C17—C18	−1.0 (4)
N1—C1—C2—C13	−133.62 (11)	C16—C17—C18—C19	0.2 (4)
C12—C1—C2—C13	103.06 (13)	C15—C14—C19—C18	−0.3 (2)
C6—C1—C2—C13	−12.12 (15)	C13—C14—C19—C18	178.73 (15)
N1—C1—C2—C3	−10.10 (11)	C15—C14—C19—O2	177.45 (14)
C12—C1—C2—C3	−133.42 (11)	C13—C14—C19—O2	−3.5 (2)
C6—C1—C2—C3	111.40 (11)	C17—C18—C19—C14	0.5 (3)
C20—C2—C3—C21	98.57 (13)	C17—C18—C19—O2	−177.40 (18)
C13—C2—C3—C21	−20.72 (15)	C13—C2—C20—O3	138.05 (15)
C1—C2—C3—C21	−145.34 (11)	C3—C2—C20—O3	15.04 (19)
C20—C2—C3—C4	−132.81 (11)	C1—C2—C20—O3	−97.92 (16)
C13—C2—C3—C4	107.91 (13)	C13—C2—C20—O2	−42.67 (15)
C1—C2—C3—C4	−16.72 (12)	C3—C2—C20—O2	−165.68 (11)
C21—C3—C4—N1	166.01 (10)	C1—C2—C20—O2	81.35 (14)
C2—C3—C4—N1	37.96 (13)	C4—C3—C21—C26	−22.73 (19)
N1—C1—C6—O1	55.83 (18)	C2—C3—C21—C26	99.17 (16)
C12—C1—C6—O1	175.18 (14)	C4—C3—C21—C22	156.91 (13)
C2—C1—C6—O1	−59.61 (18)	C2—C3—C21—C22	−81.19 (16)
N1—C1—C6—N2	−122.03 (12)	C26—C21—C22—C23	−0.4 (2)
C12—C1—C6—N2	−2.69 (13)	C3—C21—C22—C23	179.90 (14)
C2—C1—C6—N2	122.52 (12)	C21—C22—C23—C24	1.4 (3)
C12—C7—C8—C9	1.1 (2)	C22—C23—C24—C25	−1.0 (3)
N2—C7—C8—C9	−176.91 (14)	C22—C23—C24—C27	179.71 (17)
C7—C8—C9—C10	1.5 (2)	C23—C24—C25—C26	−0.4 (3)
C8—C9—C10—C11	−1.7 (3)	C27—C24—C25—C26	178.90 (18)
C9—C10—C11—C12	−0.7 (2)	C22—C21—C26—C25	−1.0 (2)
C10—C11—C12—C7	3.2 (2)	C3—C21—C26—C25	178.70 (15)
C10—C11—C12—C1	−179.77 (13)	C24—C25—C26—C21	1.4 (3)
C8—C7—C12—C11	−3.5 (2)	C3—C4—N1—C5	−177.27 (12)
N2—C7—C12—C11	174.86 (12)	C3—C4—N1—C1	−47.26 (13)
C8—C7—C12—C1	178.92 (13)	C12—C1—N1—C4	162.40 (10)
N2—C7—C12—C1	−2.76 (14)	C6—C1—N1—C4	−85.00 (12)
N1—C1—C12—C11	−53.82 (18)	C2—C1—N1—C4	35.44 (12)
C6—C1—C12—C11	−174.04 (13)	C12—C1—N1—C5	−67.79 (14)
C2—C1—C12—C11	64.87 (18)	C6—C1—N1—C5	44.80 (15)
N1—C1—C12—C7	123.46 (11)	C2—C1—N1—C5	165.25 (11)
C6—C1—C12—C7	3.23 (13)	O1—C6—N2—C7	−176.60 (14)
C2—C1—C12—C7	−117.86 (11)	C1—C6—N2—C7	1.27 (15)
C20—C2—C13—C14	57.15 (15)	C8—C7—N2—C6	179.11 (14)
C3—C2—C13—C14	178.55 (11)	C12—C7—N2—C6	0.90 (16)
C1—C2—C13—C14	−63.05 (16)	O3—C20—O2—C19	−176.49 (13)
C2—C13—C14—C19	−36.75 (18)	C2—C20—O2—C19	4.17 (18)
C2—C13—C14—C15	142.23 (15)	C14—C19—O2—C20	21.8 (2)
C19—C14—C15—C16	−0.5 (3)	C18—C19—O2—C20	−160.33 (14)

### Hydrogen-bond geometry (Å, °)

**Table:**

*D*—H···*A*	*D*—H	H···*A*	*D*···*A*	*D*—H···*A*
N2—H2···O1^i^	0.86	2.02	2.874 (1)	174.
C5—H5B···O3^ii^	0.96	2.59	3.407 (2)	143.

Symmetry codes: (i) −*x*+2, −*y*+2, −*z*; (ii) *x*, −*y*+3/2, *z*−1/2.
